# Preoperative paroxysmal atrial fibrillation predicts high cardiovascular mortality in patients undergoing surgical aortic valve replacement with a bioprosthesis: CAREAVR study

**DOI:** 10.1002/clc.23329

**Published:** 2020-02-05

**Authors:** Maunu Nissinen, Joonas Lehto, Fausto Biancari, Tuomo Nieminen, Markus Malmberg, Fredrik Yannopoulos, Samuli Salmi, Juhani K. E. Airaksinen, Tuomas Kiviniemi, Juha E. K. Hartikainen

**Affiliations:** ^1^ Heart Center Kuopio University Hospital and University of Eastern Finland Kuopio Finland; ^2^ Heart Center Turku University Hospital and University of Turku Turku Finland; ^3^ Department of Surgery Oulu University Hospital Oulu Finland; ^4^ Heart and Lung Center Helsinki University Hospital Helsinki Finland; ^5^ Department of Internal Medicine South Karelia Central Hospital Lappeenranta Finland; ^6^ Cardiovascular Medicine Brigham and Women's Hospital, Harvard Medical School Boston Massachusetts

**Keywords:** aortic valve replacement, bioprosthesis, mortality, paroxysmal atrial fibrillation, stroke

## Abstract

**Background:**

Preoperative permanent atrial fibrillation (AF) is associated with impaired outcome after surgical aortic valve replacement (SAVR). The impact of preoperative paroxysmal AF, however, has remained elusive.

**Purpose:**

We assessed the impact of preoperative paroxysmal AF on outcome in patients undergoing SAVR with bioprosthesis.

**Methods:**

A total of 666 patients undergoing isolated AVR with a bioprosthesis were included. Survival data was obtained from the national registry Statistics Finland. Patients were divided into three groups according to the preoperative rhythm: sinus rhythm (n = 502), paroxysmal AF (n = 90), and permanent AF (n = 74).

**Results:**

Patients in the sinus rhythm and paroxysmal AF groups did not differ with respect to age (*P* = .484), gender (*P* = .402) or CHA_2_DS_2_‐VASc score (*P* = .333). At 12‐month follow‐up, AF was present in 6.2% of sinus rhythm patients and in 42.4% of paroxysmal AF patients (*P* < .001). During follow‐up, incidence of fatal strokes in the paroxysmal AF group was higher compared to sinus rhythm group (1.9 vs 0.4 per 100 patient‐years, HR 4.4 95% Cl 1.8‐11.0, *P* = .001). Cardiovascular mortality was higher in the paroxysmal AF group than in the sinus rhythm group (5.0 vs 3.0 per 100 patient‐years, HR 1.70 95% CI 1.05‐2.76, *P* = .03) and equal to patients in the permanent AF (5.0 per 100 patient‐years).

**Conclusion:**

Patients undergoing SAVR with bioprosthesis and history of paroxysmal AF had higher risk of developing permanent AF, cardiovascular mortality and incidence of fatal strokes compared to patients with preoperative sinus rhythm. Life‐long anticoagulation should be considered in patients with a history of preoperative paroxysmal AF.

## 1 INTRODUCTION

Atrial fibrillation (AF) is the most common cardiac tachyarrhythmia. The prevalence of AF is 0.5% to 1% in general population, but increases with aging and about 10% of octogenarians have AF.^1^, ^2^, ^3^, ^4^ Permanent AF is associated with increased mortality and risk of thromboembolic complications.^5^ In particular, the risk is increased in AF patients with comorbidities such as hypertension, heart failure, coronary artery disease, and valvular heart diseases.^4^, ^6^, ^7^, ^8^, ^9^ Thus, there is still some debate whether AF is an independent predictor of adverse prognosis or whether the worse prognosis among AF patients rather reflects increased age and associated comorbidities. In addition, it is controversial whether the risk related to AF is equal in patients with paroxysmal and permanent AF. In recent guidelines the indications for permanent oral anticoagulation are the same in different types of AF.^10^

Several studies have demonstrated that preoperative permanent AF is a predictor of impaired outcome after adult cardiac surgery and transcatheter aortic valve implantation (TAVI).^7^, ^8^, ^10^, ^11^, ^12^, ^13^, ^14^, ^15^ However, the impact of preoperative paroxysmal AF in these patients is less well known. Namely, earlier studies included only patients with permanent AF^2^, ^13^, ^16^ or patients with paroxysmal and permanent AF, were pooled.^4^, ^14^, ^17^ Thus, the prognostic significance of paroxysmal AF has remained elusive.

The aim of this multicenter study was to evaluate the impact of preoperative paroxysmal and permanent AF on mortality and morbidity after isolated bioprosthetic SAVR in comparison to patients in sinus rhythm.

## 2 METHODS

The CAREAVR is a Finnish multicenter, retrospective registry (http://clinicaltrials.gov Identifier: NCT02626871) evaluating the incidence of AF, thromboembolic complications and bleeding events in patients undergoing isolated SAVR with bioprosthesis.

### 2.1 Patients and anticoagulation practice

Patient data were retrospectively reviewed from four Finnish university hospitals (Helsinki, Kuopio, Oulu, and Turku) over the period 2003 to 2014 (in Helsinki 2006‐2014). Hospital records were reviewed for 721 patients who underwent SAVR with a biological prosthesis. Patients with a history of cardiac surgery, permanent pacemaker as well as those undergoing any other concomitant major cardiac surgery procedure were excluded. In order to obtain complete data on cardiovascular events, only patients from the hospitals' catchment area were included in this registry.

The routine postoperative anticoagulation practice was oral warfarin and subcutaneous enoxaparin started in the evening of the day of surgery. Enoxaparin was continued until therapeutic INR > 2.0 was reached. Warfarin was continued for 3 months unless there was an indication for permanent anticoagulation. Antiplatelet agents were not used routinely postoperatively. Medication was at the treating physician's discretion.

### 2.2 Data collection

Patient records were individually reviewed with a standardized structured data collection protocol for preoperative, perioperative, and discharge data as well as for long‐term follow‐up events, such as AF, mortality, and causes of death.

Preoperative history of AF was reviewed from the patient records and based on 12‐lead ECG, Holter‐recording or telemonitoring. In addition, the preoperative rhythm was confirmed from a 12‐lead ECG recorded during admission. Based on this information, the patients were divided into three groups: (a) sinus rhythm group had no preoperative history of atrial fibrillation; (b) paroxysmal atrial fibrillation group had preoperatively at least one documented episode of atrial fibrillation cardioverted or with spontaneous recovery of sinus rhythm within 7 days and (c) permanent atrial fibrillation group had continuous atrial fibrillation prior to surgery. The presence of AF during follow‐up was assessed from 12‐lead ECG recorded at 3 and 12 months postoperative visits and whenever patients was admitted to hospital due to suspicion of symptomatic AF.

The risk of thromboembolic complications was assessed with the CHA_2_DS_2_‐VASc score^18^, ^19^ and the BARC classification (Bleeding Academic Research Consortium) was used to classify the bleeding outcomes.^20^

The study endpoints were death from any cause, cardiovascular death (cardiac death, death caused by stroke, and death due to bleeding), and other deaths. In Finland, data on deceased patients including the causes of death are prospectively collected into a national registry, Statistics Finland. This national registry was interrogated to retrieve the survival status and the eventual causes of death which were classified according to the World Health Organization ICD‐10 classification.

The study protocol was approved by the Ethics Committee of the Hospital District of Southwest Finland and of the National Institute for Health and Welfare. Informed consent was not required because of the retrospective, registry‐based nature of the study. The study conforms to the Declaration of Helsinki. An independent, certified third‐party data monitor checked the integrity of the data of each study site.

### 2.3 Statistical analysis

Continuous variables were reported as mean ± SD if normally distributed, and as median (inter‐quartile range) if they were skewed. Continuous variables were compared with one‐way ANOVA using LSD post hoc test and categorical variables were compared using Chi‐Square test or Fisher's test. Survival time analysis was performed the Cox proportional hazard model and expressed as incidence of events per 100 patient‐years. In addition, multivariate logistic regression analysis was performed to adjust for the differences in preoperative clinical characteristics between the sinus rhythm and paroxysmal AF groups. A *P*‐value of <.05 was considered statistically significant. Statistical analyses were conducted with SPSS software (version 22.0, SPSS, IBM SPSS Inc.).

## 3 RESULTS

A total of 666 patients undergoing isolated SAVR with a bioprosthesis were included in the final analysis. Patients were divided into three groups according to the history of preoperative rhythm: sinus rhythm (n = 502), paroxysmal AF (n = 90), and permanent AF (n = 74). The mean follow‐up time was 4.9 ± 2.7 years.

In the baseline clinical characteristics the paroxysmal AF group had larger left atrium diameter (*P* < .001), lower estimated glomerular filtration rate (eGFR) (*P* = .014), higher INR (*P* < .001), and they were more often on warfarin therapy (*P* < .001) compared to the sinus rhythm group (Table 1). With respect to other baseline characteristics, including age, sex, and CHA_2_DS_2_‐VASc score, patients in the paroxysmal AF group and sinus rhythm group did not differ.

**Table 1:** Preoperative clinical characteristics

	Sinus rhythm	Paroxysmal	*P*	Permanent	*P*
	(n = 502)	AF (n = 90)		AF (n = 74)	
Age	74.8 ± 6.4	75.3 ± 6.8	.484	77.6 ± 5.1	<.001*
Male	194 (38.6)	39 (43.3%)	.402	45 (60.8%)	<.001*
BMI (kg/m^2^)	27.6 ± 4.8	26.0 ± 3.9	.060	28.9 ± 5.2	.204
CHA_2_DS_2_‐VASc score	3.9 ± 1.5	4.1 ± 1.6	.333	4.6 ± 1.53	<.001*
CHADS_2_ score	2.1 ± 1.3	2.3 ± 1.2	.345	3.0 ± 1.2	<.001*
NYHA class			.918		.755
1	70 (13.9%)	11 (12.2%)		10 (13.5%)	
2	183 (36.5%)	32 (35.6%)		25 (33.8%)	
3	218 (43.4%)	40 (44.4%)		32 (43.2%)	
4	31 (6.2%)	7 (7.8%)		7 (9.5%)	
Preoperative laboratory values					
eGFR (mL/min)	75.8 ± 20.8	69.9 ± 22.2	.014*	69.8 ± 22.4	.023*
INR	1.1 ± 0.2	1.4 ± 0.7	<.001*	1.9 ± 0.5	<.001*
Medication					
Beta Blockers	277 (55.2%)	73 (81.1%)	<.001*	64 (86.5%)	<.001*
ACEI/ARB	250 (49.8%)	38 (42.2%)	.174	43 (58.1%)	.193
Digoxin	6 (1.2%)	11 (12.2%)	<.001*	29 (39.2%)	<.001*
Warfarin	15 (3.0%)	37 (41.1%)	<.001*	68 (91.9%)	<.001*
Acetylsalicylic acid	287 (57.2%)	45 (50.0%)	.223	7 (9.5%)	<.001*
ADP inhibitor	7 (1.4%)	2 (2.2%)	.631	1 (1.4%)	1.000
NSAID	13 (2.6%)	3 (3.3%)	.722	0 (0.0%)	.390
Comorbidities					
COPD	89 (17.7%)	16 (17.8%)	.996	15 (20.3%)	.569
CAD	111 (22.1%)	24 (26.7%)	.343	24 (32.4%)	.050
Diabetes	90 (17.9%)	15 (16.7%)	.767	21 (28.4%)	.034*
Dyslipidemia	277 (55.2%)	57 (63.3%)	.162	36 (48.6%)	.276
Heart failure	210 (41.8%)	37 (41.1%)	.970	45 (60.8%)	.002
Hypertension	255 (50.8%)	71 (78.9%)	.079	65 (87.8%)	.002*
PAH	108 (21.5%)	28 (31.1%)	.104	41 (55.4%)	<.001*
PAD	25 (5.0%)	4 (4.4%)	.816	6 (10.8%)	.271
Stroke or TIA	70 (13.9%)	12 (13.3%)	.918	12 (16.2%)	.508
MI	25 (5.0%)	8 (8.9%)	.137	6 (8.11%)	.264
DVT	11 (2.2%)	4 (4.4%)	.210	0 (0.0%)	.082
AE	3 (0.6%)	0 (0.0%)	1.00	0 (0.0%)	1.00
PE	7 (1.4%)	1 (1.1%)	.839	2 (2.7%)	.325
Echocardiogram					
LVEF (%)	60.6 ± 11.5	59.4 ± 14.1	.476	55.2 ± 14.1	.003*
LA (mm)	41.5 ± 6.5	44.8 ± 7.9	<.001*	50.5 ± 8.6	<.001*
AV gradient (mmHg)	81.8 ± 22.7	80.1 ± 19.3	.516	81.0 ± 24.4	.800
AR	263 (52.4%)	56 (62.2%)	.124	40 (54.1%)	1.00
MR	262 (52.2%)	54 (60.0%)	.196	52 (70.3%)	.005*

*Note*: Values are mean + SD or n (%).

Abbreviations: ACEI, angiotensin‐converting‐enzyme inhibitor; ADP, inhibitor adenosine inhibitor; AE, arterial embolism; AR, aortic valve regurgitation; ARB, angiotensin II receptor blocker; AV, aortic valve; BMI, body mass index; CAD, coronary artery disease; COPD, chronic obstructive pulmonary disease; DVT, deep venous thromboembolism; eGFR, estimated glomerular filtration rate; INR, international normalized ratio; LA, left atrium; LVEF, left ventricular ejection fraction; MI, myocardial infarction; MR, mitral valve regurgitation; NSAID, non‐steroidal anti‐inflammatory drug; NYHA, New York Heart Association Functional Classification; PAD, peripheral arterial disease; PAH, pulmonary artery hypertension; PE, pulmonary embolism.

* *P* < .05.

Patients with permanent AF were older (*P* < .001), had a higher prevalence of male gender (*P* < .001), diabetes (*P* = .034), hypertension (*P* = .002), pulmonary hypertension (*P* < .001), heart failure (*P* = .002), mitral valve regurgitation (*P* = .005), lower left ventricular ejection fraction (*P* = .003), larger left atrium diameter (*P* < .001), lower eGFR (*P* = .023), higher INR (*P* < .001), and higher CHA_2_DS_2_‐VASc score (*P* < .001) than the sinus rhythm patients (Table 1). In addition, the permanent AF patients were more often on warfarin (*P* < .001), beta blocker (*P* < .001), and digoxin (*P* < .001) medication as well as used aspirin less frequently than the sinus rhythm patients (Table 1).

### 3.1 Late outcome

**Figure 1:**
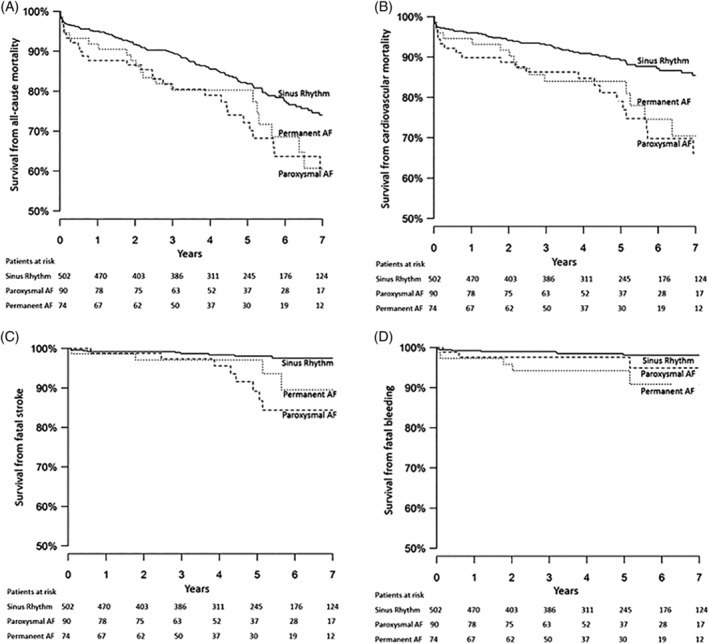
Kaplan‐Meier curves for survival from: A, all‐cause mortality; B, cardiovascular mortality; C, fatal stroke; and D, fatal bleeding in patients undergoing isolated bioprosthetic surgical aortic valve replacement. AF, atrial fibrillation

**Figure 2:**
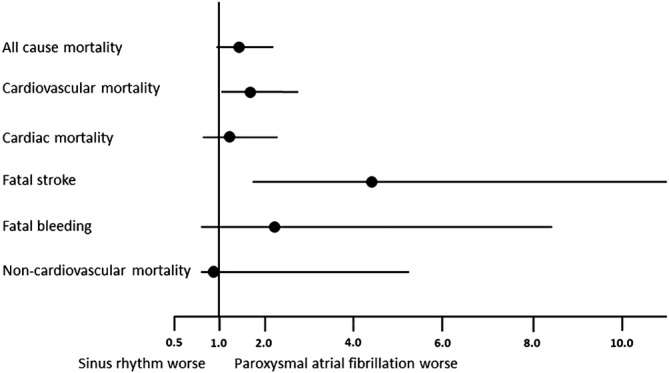
Outcomes in patients with preoperative paroxysmal atrial fibrillation vs preoperative sinus rhythm (hazard ratios and 95% confidence intervals)

All‐cause mortality in the paroxysmal AF group (7.2 per 100 patient‐years at risk) tended to be higher compared to the sinus rhythm group (4.9 per 100 patient‐years) (HR 1.5, 95% CI 1.0‐2.2, *P* = .057) (Table 2; Figures 1A and 2). The Kaplan‐Meier estimates of survival in the paroxysmal AF and sinus rhythm groups at 1, 3, and 5 years were 87.8%, 82.2%, 75.6% and 95.0%, 89.8%, 84.3%, respectively (Figure 1A). All‐cause mortality in the permanent AF group (7.1 per 100 patient‐years) also tended to be higher than in the sinus rhythm group (HR 1.6, 95% CI 1.0‐2.4, *P* = .055) with Kaplan Meier estimates of survival at 1, 3, and 5 years of 91.9%, 81.1%, 81.1%, respectively.

**Table 2:** Long‐term follow‐up data

Mortality	Sinus rhythm	Paroxysmal AF		Permanent AF	
	n = 502	n = 90	*P*‐value (HR, 95% CI)	n = 74	*P*‐value (HR, 95% CI)
All cause	125 (24.9) [4.9]	30 (33.3) [7.2]	.057 (HR 1.5, 95% Cl 1.0‐2.2)	23 (31.1) [7.1]	.055 (HR 1.6, 95% Cl 1.0‐2.4)
1 year*	95.0%	87.8%		91.9%	
3 year*	89.8%	82.2%		81.1%	
5 year*	84.3%	75.6%		81.1%	
Cardiovascular	75 (14.9) [3.0]	21 (23.3) [5.0]	.032 (HR 1.7, 95% Cl 1.1–2.8)	16 (21.6) [5.0]	.039 (HR 1.8, 95% Cl 1.0‐3.1)
1 year*	96.0%	90%		94.6%	
3 year*	93.2%	86.7%		85.1%	
5 year*	90.6%	82.2%		85.1%	
Cardiac	53 (10.6) [2.1]	11 (12.2) [2.6]	.506 (HR 1.3, 95% Cl 0.7‐2.4)	9 (12.2) [2.8]	.355 (HR 1.4, 95% Cl 0.7‐2.8)
Stroke	11 (2.2) [0.4]	8 (8.9) [1.9]	.001 (HR 4.4, 95% Cl 1.8–11.0)*	4 (5.4) [1.2]	.076 (HR 2.8, 95% Cl 0.9‐8.9)
1 year*	99.2%	98.9%		98.6%	
3 year*	98.8%	97.8%		97.3%	
5 year*	98.4%	93.3%		97.3%	
Bleeding	9 (1.8) [0.4]	3(3.3) [0.7]	.234 (HR 2.2, 95% Cl 0.6‐8.5)	5 (6.8) [1.6]	.005 (HR 5.2 95% Cl 1.6–16.2)
1 year*	99.2%	97.8%		97.3%	
3 year*	99.0%	97.8%		94.6%	
5 year*	98.4%	97.8%		94.6%	
Other vascular	6 (1.2) [0.2]	1 (1.1) [0.2]	.919 (HR 1.1, 95% Cl 0.1‐9.3)	1 (1.4) [0.3]	.662 (HR 1.6, 95% Cl 0.2‐13.5)
Non‐cardiovascular	50 (10.0] [2.0]	9 (10.0) [2.2]	.752 (HR 1.1, 95% Cl 0.6‐2.3)	7 (9.5) [2.2]	.649 (HR 1.2, 95% Cl 0.5‐2.7)
Cancer	28 (5.6) [1.1]	4 (4.4) [1.0]	.802 (HR 0.9, 95% Cl 0.3‐2.5)	3 (4.1) [0.9]	.905 (HR 0.9, 95% Cl 0.3‐3.1)
Other	3 (0.6) [0.1]	0 (0.0) [0.0]	.658 (HR 0.8, 95% Cl 0.04‐15.4)	1 (1.4) [0.3]	.403 (HR 2.6, 95% Cl 0.3‐25.3)

*Note*: Values are n (%) and [incidence per 100 patient‐years].

Abbreviations: AF, atrial fibrillation; HR, hazard ratio.

* Denotes survival.

Cardiovascular mortality in the paroxysmal group (5.0 per 100 patient‐years) was significantly higher compared to the sinus rhythm group (3.0 per 100 patient‐years) (HR 1.7 95% Cl 1.1‐2.8, *P* = .032) (Table 2; Figures 1B and 2). In particular, mortality from fatal stroke was 1.9 per 100 patient‐years in the paroxysmal AF patients as compared to 0.4 per 100 patient‐years in the sinus rhythm patients (HR 4.4 95% Cl 1.8‐11.0, *P* = .001) (Figures 1C and 2). Cardiovascular mortality was also higher in the permanent AF group (5.0 per 100 patient‐years) than in the sinus rhythm group (HR 1.8 95% Cl 1.0‐3.1, *P* = .039). This was mainly due to high rate of fatal bleeds 1.6 per 100 patient‐years compared to 0.3 per 100 patient‐years in the sinus rhythm patients (HR 5.2 95% Cl 1.6‐16.2, *P* = .005 (Figure 1D).

Logistic regression analysis was also performed to adjust for the differences in preoperative clinical characteristics (eGFR and left atrium size) between the sinus rhythm and paroxysmal AF groups. After adjustment of these, paroxysmal AF remained as an independent predictor of fatal stoke (HR 3.4, 95% CI 1.2‐9.8, *P* = .025). However, paroxysmal AF lost its significance as predictor of cardiovascular mortality (HR 1.7, 95% CI 0.9‐3.1, *P* = .126).

The majority of fatal strokes were of ischemic origin (81.8% in the sinus rhythm group, and 75.0% in the paroxysmal and permanent AF groups). The incidence of fatal ischemic strokes in the paroxysmal AF group was higher compared to the sinus rhythm group (1.4 vs 0.4 per 100 patient years, HR 4.1, 95% CI 1.4‐11.4, *P* = .008). There was also a trend (albeit non‐significant) of higher incidence of fatal hemorrhagic strokes in the paroxysmal AF group (0.5 vs 0.1 per 100 patient years, HR 6.0, 95% CI 0.8‐42.7, *P* = .07).

The time to first stroke/TIA were 4.4 + 2.8 years for the sinus rhythm group, 4.1 + 3.0 years for the paroxysmal AF group (*P* = .402 vs sinus rhythm group), and 3.5 + 2.7 years for the permanent AF group (*P* = .017 vs sinus rhythm group). The time to the first major bleed were 4.9 + 2.8 years in the sinus rhythm group, 4.2 + 3.0 years for the paroxysmal AF group (*P* = .073 vs sinus rhythm group), and 3.7 + 2.7 years for the permanent AF group (*P* = .004 vs sinus rhythm group).

At discharge, 3‐ and 12‐months after surgery, AF was present in 38.9%, 36.6%, and 42.4% of patients in the paroxysmal AF group and in 17.3%, 10.8%, and 6.2% of patients in the sinus rhythm group, respectively (*P* < .001 for all) (Table 3). In the permanent AF group, AF was detected in 90.5%, 85.2%, and 88.9% (*P* < .001 for all vs sinus rhythm). Furthermore, AF (paroxysmal or permanent) was documented in 64% of the paroxysmal AF group patients during the 12‐month follow‐up after the discharge.

**Table 3:** Rhythm status, warfarin, and aspirin medication during follow‐up

	Sinus rhythm	Paroxysmal AF		Permanent AF	
	n = 502	n = 90	*P*‐value	n = 74	*P*‐value
Rhythm = AF					
At discharge	87 (17.3)	35 (38.9)	<.001	67 (90.5)	<.001
At 3 months	54 (10.8)	26 (36.6)	<.001	52 (85.2)	<.001
At 12 months	31 (6.2)	14 (42.4)	<.001	40 (88.9)	<.001
Medication					
Warfarin after 3 months	87 (31.9)	42 (59.2)	<.001	59 (98.3)	<.001
Warfarin after 12 months	65 (35.9)	22 (50.0)	.085	45 (97.8)	<.001
2003‐8/2006	13 (37.1)	2 (40.0)		1 (100.0)	
9/2006‐5/2010	27 (35.5)	14 (53.8)		19 (100.0)	
6/2010‐2014	25 (35.7)	6 (46.2)		25 (96.2)	
ASA after 3 months	141 (51.8)	18 (25.4)	<.001	4 (6.6)	<.001
ASA after 12 months	94 (52.5)	16 (36.4)	.055	3 (6.7)	<.001

*Note*: Values are n (%).

Abbreviations: AF: Atrial fibrillation; ASA: aspirin.

All patients were on warfarin therapy at 3‐month follow‐up visit. Thereafter warfarin was continued in 59.2% of the paroxysmal AF patients and in 31.9% of the sinus rhythm patients (*P* < .001) (Table 3). At 12‐month follow‐up, 50% patients in the paroxysmal AF patients and 35.9% in the sinus rhythm patients were on warfarin therapy with borderline difference between the groups (*P* = .085). In the permanent AF group 98.3% and 97.8% of patients were on warfarin at 3 and 12 months follow‐up. There was no significant change in the use of oral anticoagulation during the study period in the paroxysmal AF patients.

Of the patients suffering from fatal stroke, 87.5% in the paroxysmal AF, 14.2% in the sinus rhythm, and 75% in the permanent AF group were on OAC at the time of event. However, only 14.2% of the deceased patients in the paroxysmal AF group had INR within the target range (2.0‐3.0). In the sinus rhythm and permanent AF groups none of the patients had INR within the therapeutic range. In sinus rhythm and permanent AF groups there were one patient in both groups without INR value taken within 1 month preceding the event.

At 3‐month follow‐up visit, permanent aspirin treatment was prescribed in 25.4% of the paroxysmal AF patients and 51.8% of the sinus rhythm patients (*P* < .001). At 12‐month follow‐up aspirin was used in 36.4% patients in the paroxysmal AF group and 52.5% in the sinus rhythm group (*P* = .055). In the permanent AF group aspirin was prescribed in 6.6% and 6.7% of patients at 3‐ and 12‐month follow‐up, respectively (*P* < .001 for both vs sinus rhythm group) (Table 3).

## 4 DISCUSSION

The main findings of the study were that patients with preoperative paroxysmal AF undergoing SAVR had high risk of developing permanent AF during follow‐up. In addition, they were at higher risk of cardiovascular death than patients with sinus rhythm, the risk being similar to patients with permanent AF. The increased mortality in paroxysmal AF patients was mainly driven by fatal strokes. At the time of fatal stroke, INR was not within the therapeutic range in a great majority of patients.

The finding of high risk of permanent AF and fatal strokes during follow‐up in patients with preoperative paroxysmal AF has pivotal clinical implications in patient care. The data suggest that patients with preoperative paroxysmal AF need to be considered as candidates for life‐long oral anticoagulation after bioprosthetic SAVR irrespectively of their postoperative rhythm. Given the well‐defined therapeutic efficacy of oral anticoagulation therapy in permanent and paroxysmal AF,^21^, ^22^ the current guidelines recommend anticoagulation in patients with permanent and paroxysmal AF and risk factors for stroke.^10^ In our study only a half of the patients with preoperative paroxysmal AF were on oral anticoagulation therapy 12 months postoperatively. Considering that 42% of patients with preoperative paroxysmal AF were in AF at 12‐month follow‐up, it seems that anticoagulation was prescribed only in paroxysmal AF patients developing permanent AF during follow‐up. Thus, the treatment of paroxysmal AF patients, particularly those with a high risk of developing permanent AF, seems to be far from optimal. In line with the present findings, recently Mogensen et al reported that 53% of their heart failure patients with paroxysmal AF were on anticoagulation.^12^ Palomäki et al evaluated patients with acute stroke or transient ischemic attack and a history of AF.^23^ Of those that were anticoagulation naive at time of the event, 50% had a history of paroxysmal AF. The high incidence of permanent AF and the high risk of fatal stroke in patients with a history of preoperative paroxysmal AF undergoing bioprosthetic SAVR highlights the importance of life‐long anticoagulation in these patients.

Importantly, cardiovascular mortality in the paroxysmal AF patients was significantly higher compared to those in sinus rhythm and of same magnitude as in patients with permanent AF. This was somewhat surprising since as comes to the preoperative clinical characteristics patients with paroxysmal AF were well matched with their counterparts in sinus rhythm. These two groups did not differ with respect cardiovascular risk factors such as cardiac function, CHA_2_DS_2_‐VASc score or other comorbidities, which are significant predictors of mortality and morbidity in cardiac patients and patients with AF as well as also patient with bioprosthesis.^24^ Paroxysmal AF group had lower eGFR and larger left atrium diameter. However, after adjustment of these, paroxysmal AF remained as an independent predictor of fatal strokes.

In the present study fatal strokes were primarily responsible for the high cardiovascular mortality in the paroxysmal AF patients with 4‐fold risk compared to the sinus rhythm patients and 1.5‐fold compared to permanent AF patients. On the other hand, there was no significant difference between patients with paroxysmal AF and sinus rhythm with respect to cardiac deaths or non‐cardiac deaths. Stroke and other thromboembolic complications in AF patients are often of embolic origin.^25^ Correspondingly, our results suggest that AF resulting in cardio‐embolism rather than heart failure or sudden death were responsible for the increased mortality among patients with paroxysmal AF. Our results are also in line with a Danish study including 15 000 AF patients with heart failure.^12^ They reported that patients with paroxysmal AF had higher risk of stroke than patients with sinus rhythm or persistent or permanent AF. These findings suggest that in spite of an increased risk of thromboembolism, anticoagulation therapy has not been adopted properly in patients with paroxysmal AF undergoing SAVR with a bioprosthesis.

What merits to be addressed is that only 14% of the paroxysmal AF group patients suffering from fatal stroke and prescribed OAC had INR preceding the event within the target range. Further, in the permanent AF group none had INR within the therapeutic range at the time of bleeding event. These suggest that in most cases the events, whether stroke or bleeding were related to poor anticoagulation control. This is in accordance with a previous report in patients with non‐valvular AF. In AF patients on OAC and suffering from stroke, INR at the time of event was outside the therapeutic target in more than 50% of patients.^23^

Permanent AF is a well‐known predictor of adverse outcomes in patients scheduled for SAVR (14). However, to our best knowledge there are no earlier studies addressing the question of paroxysmal AF in patients undergoing SAVR with a bioprosthesis. A closest counterpart to our study is a recent paper by Shaul et al.^8^ They evaluated the differential impact of paroxysmal and permanent AF) on the outcome after TAVI. In contrast to our findings, they reported that the risk of death or stroke at 2 years was similar in patients with sinus rhythm (16%) and paroxysmal AF (15%), whereas it was significantly higher among patients with permanent AF (38%). Such differences might be explained by a different risk profile in TAVI patients compared to patients undergoing SAVR with a bioprosthesis as well as by the fact that temporary anticoagulation is not used after TAVI.^26^

Ngaage et al^11^ evaluated 252 patients with preoperative sinus rhythm, 89 patients with intermittent (paroxysmal) AF and 34 with continuous (permanent) AF. Preoperative AF (including patients with paroxysmal and permanent AF) was associated with 50% increase in late all‐cause mortality but not with cardiac death. In our study all‐cause mortality in patients with paroxysmal and permanent AF was also 50% higher compared to patients in sinus rhythm. However, in contrast to us, Ngaage et al^11^ reported a 9‐fold incidence of cardiac death in patients with permanent AF when compared to patients with paroxysmal AF whereas in our study cardiac mortality in patients with paroxysmal and permanent AF was of same magnitude. Whether the finding by Ngaage was due to differences in comorbidities between the groups cannot be addressed because the clinical characteristics of patients in the paroxysmal and permanent AF groups were not presented separately.

It is also of interest that in our study the incidence of permanent AF during postoperative follow‐up was very high among the patients with preoperative paroxysmal AF. All patients with preoperative paroxysmal AF were in sinus rhythm at the time of surgery. However, a substantial proportion (39%) was discharged in AF and at 3‐ and 12‐month follow‐up visits permanent AF was present in 37% and 42%, respectively. In addition, almost 2/3 of patients in the paroxysmal AF group had documented AF (paroxysmal of persistent) during the first 12 months after the discharge. Most probably, the incidence of AF would be even higher if an ambulatory ECG recording had been applied. The high incidence of AF is in line with Ngaage et al who reported that 50% of patients with preoperative paroxysmal AF undergoing SAVR were in permanent AF during follow‐up.^11^ In addition, we observed that in the paroxysmal AF patients, the incidence of permanent AF increased by time whereas it decreased in the patients with preoperative sinus rhythm during the follow‐up. This suggests that preoperative paroxysmal AF, even in the absence of other comorbidities, identifies a group of patients who are prone to develop permanent AF postoperatively. In addition, it seems that cardiac surgery speeds up the progression from paroxysmal to permanent AF. Mogensen et al^12^ reported that the risk of death and thromboembolic complications was highest among patients with new onset AF. The reason for this remains unclear, but a possible explanation is that patients with new onset AF received less aggressive treatment to control AF heart rate and are less often prescribed anticoagulation.

### 4.1 Strengths and limitations

This study has several methodological strengths. The data were collected from electronic patient records and hospital database in which the data are stored prospectively in predefined form. A validated, structured case report form was used. A quality control monitoring of the data was performed by a clinical research organization. The patient population comes from the hospital regional catchment area where all cerebrovascular events are treated exclusively at the participating centers. All patients undergoing SAVR were scheduled for follow‐up visits at 3 and 12 months. Thus, the follow‐up data were available from almost all patients. A major strength of our study is also that in Finland each patient has a national identification code. This allows the follow‐up and identification of patients' date and cause of death through the country. Nevertheless, the retrospective set‐up carries also some limitations. It does not allow characterization of the study populations as accurately as in a prospective trial. An obvious limitation is that the presence of AF during follow‐up was based on 12‐lead ECG recorded during routine 3‐ and 12‐month follow‐up visits or in case of symptomatic AF episodes whereas no routine Holter recordings were performed. Thus, it is likely that some episodes of asymptomatic paroxysmal AF were missed.

## 5 CONCLUSIONS

In patients scheduled for SAVR with bioprosthesis, paroxysmal AF was associated with increased mortality—particularly increased risk of fatal strokes—and higher risk of developing permanent AF compared to those with a history of sinus rhythm. Life‐long anticoagulation should be considered in these patients after the surgery.

## CONFLICT OF INTEREST

Maunu Nissinen: research grants from Rättimäki CardioVascular Society and travel grants from Finnish Cardiac Society.

Joonas Lehto: research grants from Orion Research Foundation and the Finnish Foundation for Cardiovascular Research.

Markus Malmberg: research grant from Clinical Research Fund (EVO) of Turku University Hospital, Turku, Finland.

Juha Hartikainen: research grants from the Finnish Foundation for Cardiovascular Research, Clinical Research Fund (VTR) of Kuopio University Hospital, Kuopio, Finland; Lectures for: Cardiome AG, MSD and AstraZeneca. Member of the advisory boards for Amgen, Pfizer, MSD, AstraZeneca, Bayer and BMS.

Leo Ihlberg: full‐time employee and stockholder for Boston Scientific (since September 2016); proctor for Edwards Lifesciences.

Juhani Airaksinen: research grants from the Finnish Foundation for Cardiovascular Research, Clinical Research Fund (EVO) of Turku University Hospital, Turku, Finland; lectures for Bayer, Cardiome AG and Boehringer Ingelheim, member in the advisory boards for Bayer, Astra Zeneca, Bristol‐Myers Squibb‐Pfizer and Boston Scientific.

Tuomo Nieminen: lectures for AstraZeneca, Boehringer Ingelheim, FCG Koulutus, GE Healthcare, Medtronic, Orion, Sanofi; research grants from Abbvie, Medtronic, research fund of Helsinki and Uusimaa Hospital District.

Tuomas Kiviniemi: lectures for Bayer, Boehringer Ingelheim, Medicines Company, AstraZeneca and St. Jude Medical, Bristol‐Myers Squibb‐Pfizer, MSD; received research grants from The Finnish Medical Foundation, Helsinki, Finland; the Finnish Foundation for Cardiovascular Research; Clinical Research Fund (EVO) of Turku University Hospital, Turku, Finland, Finnish Cardiac Society, an unrestricted grant from Bristol‐Myers Squibb‐Pfizer; Emil Aaltonen Foundation, Helsinki, Finland; and Maud Kuistila Foundation, Helsinki, Finland; member of advisory board for Boehringer Ingelheim, MSD.
